# Brain metabolomic profiling of eastern honey bee (*Apis cerana*) infested with the mite *Varroa destructor*

**DOI:** 10.1371/journal.pone.0175573

**Published:** 2017-04-12

**Authors:** Jiang-Li Wu, Chun-Xue Zhou, Peng-Jie Wu, Jin Xu, Yue-Qin Guo, Fei Xue, Awraris Getachew, Shu-Fa Xu

**Affiliations:** 1 Key Laboratory of Pollinating Insect Biology, Ministry of Agriculture, Institute of Apicultural Research, Chinese Academy of Agricultural Sciences, Beijing, China; 2 Department of Parasitology, Shandong University School of Basic Medicine, Jinan, Shandong Province, PR China; 3 National Animal Protozoa Laboratory and College of Veterinary Medicine, China Agricultural University, Beijing, PR China; Monash University, AUSTRALIA

## Abstract

The mite *Varroa destructor* is currently the greatest threat to apiculture as it is causing a global decrease in honey bee colonies. However, it rarely causes serious damage to its native hosts, the eastern honey bees *Apis cerana*. To better understand the mechanism of resistance of *A*. *cerana* against the *V*. *destructor* mite, we profiled the metabolic changes that occur in the honey bee brain during *V*. *destructor* infestation. Brain samples were collected from infested and control honey bees and then measured using an untargeted liquid chromatography-tandem mass spectrometry (LC-MS/MS)-based global metabolomics method, in which 7918 and 7462 ions in ESI+ and ESI- mode, respectively, were successfully identified. Multivariate statistical analyses were applied, and 64 dysregulated metabolites, including fatty acids, amino acids, carboxylic acid, and phospholipids, amongst others, were identified. Pathway analysis further revealed that linoleic acid metabolism; propanoate metabolism; and glycine, serine, and threonine metabolism were acutely perturbed. The data obtained in this study offer insight into the defense mechanisms of *A*. *cerana* against *V*. *destructor* mites and provide a better method for understanding the synergistic effects of parasitism on honey bee colonies.

## Introduction

Honey bees provide pollination services to a diverse array of agricultural crop plants, which is a highly valued resource around the world [1]. The recent sharp decline in honey bee populations has caused a global crisis as the honey bee supply cannot keep up with the increase in agricultural demands [2]. Possible causes of the declines include exposure to certain pesticides, diseases, parasites, and even environmental deterioration [3]. Worth mentioning, the parasitic mite, *Varroa destructor*—originally confined to the eastern honey bee, *Apis cerana—*has become the most detrimental parasite of the western honey bee, *Apis mellifera*, and currently is considered as the major threat to apiculture [2].

The factors by which *V*. *destructor* cause honey bee declines have been widely studied and have been attributed to two main causes: 1) female mites and their offspring, which feed on bee hemolymph for reproduction, weakening the honey bee’s immune system, and 2) mites, which serve as an active vector of pathogenic viruses, and make the bees become more vulnerable to other secondary pathogens [4–6]. Previous studies have shown that all honey bee colonies are infested with *Varroa* mites. Unless steps are taken to reduce mite levels, colonies usually die within six months to two years. Meanwhile, infested bee species often exhibit dwindling populations and symptoms of viral and brood diseases [7].

Colonies of the Asian honey bee *A*. *cerana* are more resistant to *V*. *destructor* and suffer less damage than the western honey bee *A*. *mellifera*. Several effective defense strategies have been implicated such as grooming and hygienic behaviors [8]. Grooming behavior is regarded as the most important behavioral trait contributing to the defense against the parasitic mite. It consists of self-cleaning, a grooming dance, nestmate cleaning, and group cleaning [9]. Understanding the resistance mechanisms of the eastern honey bees against *Varroa* mites will help in breeding hybrids or other bees that are resistant to *Varroa destructor*. Dopamine, an important neurotransmitter in the brain, produces significant shifts in honey bee grooming behaviors and functions in a dose- and time-dependent manner [10]. Hygienic behavior is affected by olfactory cues such as by the odor of diseased, parasitized, or dead broods. The neuromodulator octopamine has been shown not only to enhance the response of bees to olfactory stimuli but also to play a role in biasing the nervous system for behavioral shaping [11]. Tyramine acts as the biosynthetic intermediate precursor for octopamine, which is capable of being released from neurons and influencing the honey bee’s locomotor activity [12]. Taken together, these findings suggest that behavioral changes may be caused by neuropathological and neurophysiological effects on the honey bee’s central nervous system.

Metabonomics is defined as the comprehensive quantitative and qualitative analysis of all metabolites in cells, tissues, or biofluids, and is as an emerging “-omics” method that provides surprisingly detailed insights into various metabolic processes under certain physiological or pathological conditions [13,14]. In the present study, a metabolomic analysis based on LC-Q-TOF MS/MS was first used to gain a better understanding of metabolic profile differences of *A*. *cerana* worker bee’s brains before and after *V*. *destructor* infestation. The differentially expressed metabolites identified in this study will further increase our understanding of the resistance mechanisms of eastern bees against *Varroa* mites. Meanwhile, the data obtained in this study will also provide information likely to be critical for the development of rationally designed *V*. *destructor* prevention methods and for the breeding of new mite-tolerant honey bees.

## Results

### Grooming behavior at the individual level

**Fig 1:**
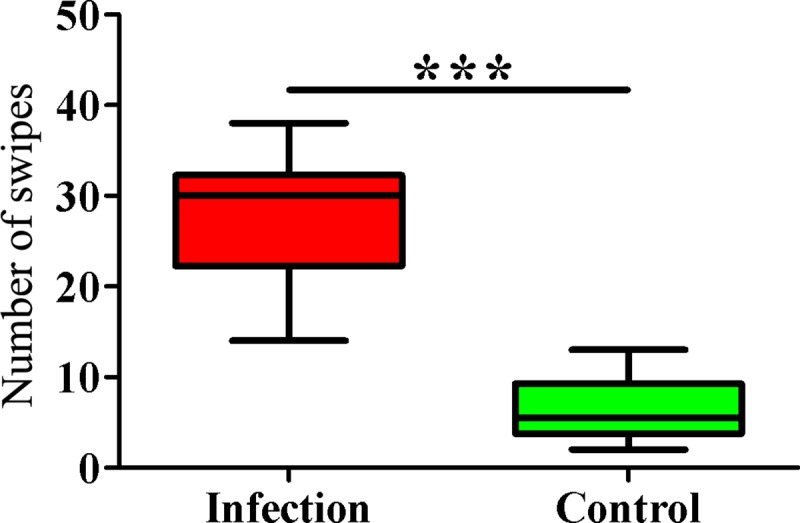
Behavioral observation results. *** indicates *p* < 0.001.

Mite resistance is considered a typical trait of eastern honey bees, and grooming is an important mechanism of protection against mite invasion. To determine grooming behavior changes during *V*. *destructor* infestation, both infested and non-infested bees were observed in a small modified Plexiglas^®^ cage for 5 min, and individual grooming behaviors were recorded. As shown in Fig 1, the number of swiping behavior was extremely significantly higher in the *V*. *destructor* infection group compared to the control (*p* <0.001).

### Analysis of brain metabolic patterns from infested and non-infested bees

**Fig 2:**
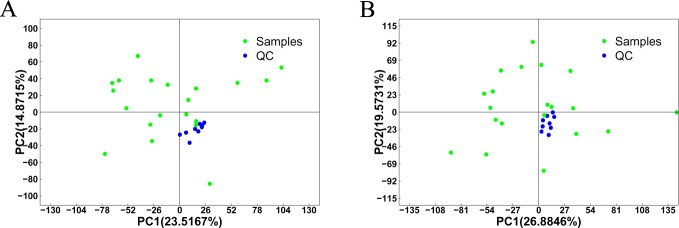
PCA of QC samples. (A) Score plot for the positive ion mode; (B) Score plot for the negative ion mode.

**Fig 3:**
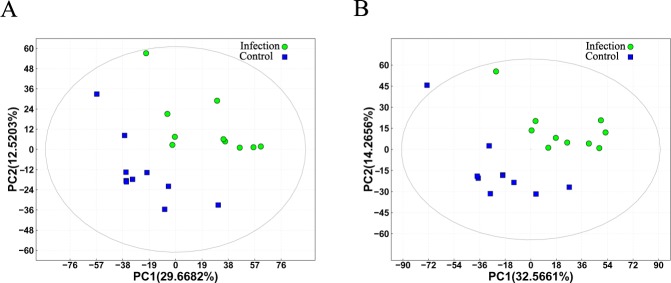
PLS-DA score plot of *Varroa destructor*-infested (Infection) and control (Control) bees. (A) Score plot for the positive ion mode (R^2^ = 0.8102, Q^2^ = 0.2513); (B) Score plot for the negative ion mode (R^2^ = 0.8151, Q^2^ = 0.3466). In the score plot, each data point represents one bee brain sample, and the distance between points indicates the similarity between samples. x- and y-axes represent PC1 and PC2, respectively.

To assess the capability of the LC-MS/MS-based metabolomic approach to differentiate *V*. *destructor*-infested bees from controls, we first analyzed all total ion chromatograms of honey bee brains. As shown in S1 Fig, the total ion chromatograms exhibited a stable retention time without obvious peaks’ drifts. There were 8568 and 8210 ions identified in each sample profile under the ESI+ and ESI- mode, respectively. Nine QC samples were run for bee brain samples throughout the analysis. As shown in Fig 2, QC samples clustered together in the principal component analysis (PCA), which indicated that the LC-MS/MS system exhibited high stability and reproducibility. After removing low-quality ions (relative standard deviation (RSD) > 30%), we identified 7918 and 7462 ions in each sample analyzed in ESI+ or ESI- mode, respectively. To better illustrate the metabolic variations in honey bee brain after infestation by *V*. *destructor*, multivariate PCA and partial least squares discriminant analyses (PLS-DA) were applied to process the data. The plots of PCA scores showed no obvious separation between the infected group and the non-infected group in both ESI+ and ESI- modes (S2 Fig). Subsequently, the PLS-DA models used in further multivariate analyses exhibited good separation and are shown in Fig 3.

**Fig 4:**
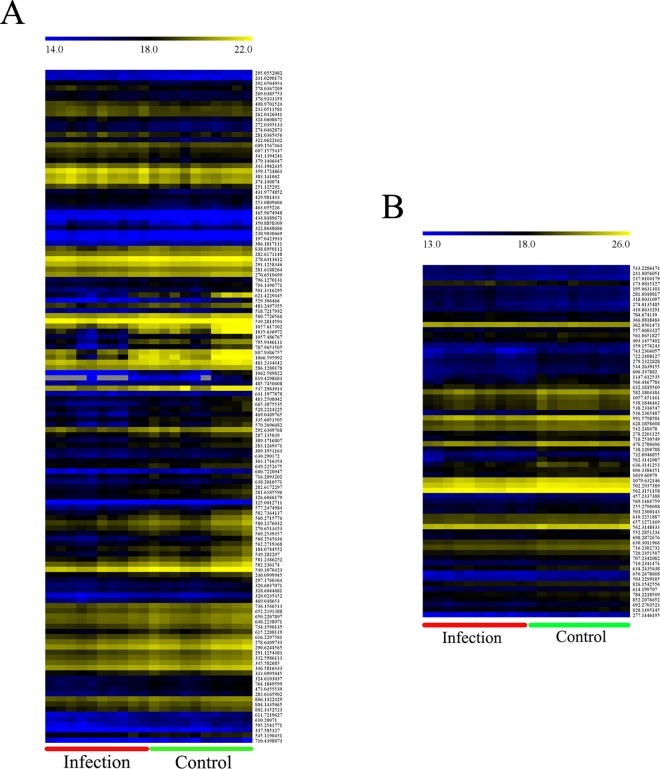
Comparison of the bee brain metabolomes between infested and control groups. Aheat map was generated using the open source analysis software MeV v.4.9 (http://www.tm4.org/mev/MeV_4_6) to show the log_2_ metabolite abundances between the infection and control groups. (A) Heat map of metabolitesdetected in positive ion mode; (B) Heat map of metabolites detected in negative ion mode.

Pairwise comparisons of abundances revealed ions in the infected group that were statistically differentially expressed from those in the control (corrected *p*-value < 0.05, FC > 1.2 or < 0.8). We constructed heat maps (commonly used for unsupervised clustering) based on these significantly different metabolites, which did not exhibit obvious segregation. In the ESI+ mode, 130 ions differentiated the infection group from the controls (Fig 4A), and in the ESI- mode, 68 differentially expressed ions were detected (Fig 4B).

### Metabolic pathway analysis

We found putative identifications for 64 ion masses (all positive and negative mode ions) by searching against the mass-based HMDB database. Table 1 present the putative metabolites detected in ESI+ and ESI- mode, respectively. In the ESI+ mode, most identified metabolites were down-regulated. Among the down-regulated metabolites, iriomoteolide-1a was the least abundant metabolite (FC = 0.16). Meanwhile, more than half of the metabolites identified in the ESI- mode were less abundant. Metabolites like nizatidine, 2-propylglutaric acid, chlorphenesin, glycine, fonofos, and GMP were more abundant.

**Table 1:** List of differentially-expressed metabolites identified in ESI+ and ESI- mode.

Mode	m/z	Rt (min)	VIP	FC	*q*-value	Formula	Name
ESI+	341.1394	4.6283	1.368351	1.285	0.036	C20H18N2O	Nicofetamide
	292.0505	4.55875	1.596902	1.397	0.036	C11H11NO6	N-Pyruvoyl-5-methoxy-3-hydroxyanthranilate
	278.0367	4.55875	1.598379	1.382	0.036	C9H7N7O2S	Azathioprine
	806.1422	7.465567	1.544685	0.731	0.036	C24H40N7O17P3S	Propanoyl-CoA
	283.1269	7.867833	1.42322	0.749	0.041	C12H15N3O2S	Albendazole
	650.2208	7.465567	1.490081	0.739	0.036	C27H37ClFN3O12	Mosapride citrate hydrate
	383.1411	4.65815	1.673014	1.514	0.044	C24H24O2	alpha,alpha'-Diethyl-4,4'-bis(2-propynyloxy)stilbene
	197.0424	4.383483	1.375502	1.292	0.036	C7H10O5	Shikimate
	322.0622	4.542683	1.718128	1.533	0.041	C16H13ClFNO3	Flamprop
	431.9775	4.542683	1.6817	1.473	0.036	C13H7Cl2F3N2O4S	Flusulfamide
	241.029	4.55875	1.611376	1.387	0.036	C12H10O2S	cis-1,2-Dihydroxy-1,2-dihydrodibenzothiophene
	184.0745	7.465567	1.995063	0.566	0.036	C5H11O4P	Isopentenyl phosphate
	282.6172	7.465567	1.747623	0.654	0.036	C26H37N5O5S2	UR-12947
	283.6166	7.465567	1.75264	0.647	0.037	C27H35NO12	Ipecoside
	501.3116	7.465567	2.043752	0.535	0.042	C33H38N6	Hodgkinsine
	529.3065	7.465567	2.911493	0.307	0.036	C16H19N3	Fenapanil
	600.7221	7.465567	1.980842	0.570	0.036	C48H80N7O20P3S	(25S)-3alpha,7alpha,12alpha-Trihydroxy-5beta-cholestan-26-oyl-CoA
	320.0235	7.465567	1.901228	0.600	0.039	C16H11Cl2NO2	2,6-Dichlorophenol-4-(1,4-naphthoquinone imine)
	291.1258	7.569433	1.239254	0.815	0.036	C16H19ClN2O	Carbinoxamine
	1035.637	7.569433	3.58705	0.160	0.037	C29H46O7	Iriomoteolide 1a
	481.2334	7.583717	1.779418	0.652	0.036	C27H34N2O7	Moexipril
	1057.487	7.598017	2.383582	0.469	0.046	C29H36O9	Satratoxin H
	469.049	7.853383	2.372853	0.500	0.037	C11H20N4O11P2	CDP-ethanolamine
	309.1951	7.853383	1.432012	0.754	0.036	C20H26N2O2	Hydroquinidine
	630.2902	7.867833	1.734141	0.671	0.036	C33H45NO12	Beiwutine
	389.1717	7.853383	1.90607	0.600	0.037	C20H30O5	Prostaglandin D3
	483.2497	7.853383	1.951121	0.594	0.036	C30H36O4	Sophoranone
	665.3876	7.465567	2.665727	0.387	0.036	C36H56O11	Phytolaccoside B
	297.176	7.465567	1.833047	0.623	0.036	C5H12N2O3	N5-Hydroxy-L-ornithine
	291.1254	7.465567	1.678301	0.668	0.036	C16H19ClN2O	Carbinoxamine
	240.1	7.465567	1.639634	0.687	0.036	C13H15NO2	Securinine
	251.1253	5.261983	1.646936	1.446	0.036	C12H20O4	Traumatic acid
	374.1401	4.67295	1.605937	1.460	0.046	C18H26ClN3O	Hydroxychloroquine
	295.0552	4.643267	1.276685	1.270	0.043	C15H12O5	Pinobanksin
	272.0395	4.55875	1.788028	1.514	0.036	C14H11NO4S	N-(6-Oxo-6H-dibenzo[b,d]pyran-3-yl)methanesulfonamide
	274.0463	4.55875	1.664588	1.455	0.036	C15H9NO3	7-Methylpyrido[3,4-c]psoralen
	324.0609	4.542683	1.646956	1.465	0.036	C15H15BrO2	Monobromobisphenol A
	253.081	1.26065	1.603063	1.411	0.049	C11H14N2O6	Clitidine
	238.9031	0.519167	1.686938	1.530	0.043	C2H5Br	Bromoethane
ESI-	362.0501473	0.9973	1.304551935	1.256	0.047	C10H14N5O8P	GMP
	826.1542556	7.462816667	1.669148855	0.712	0.024	C36H37O20	Delphinidin 3-glucoside 5-caffoyl-glucoside
	403.1457402	7.234233333	1.651665788	0.698	0.042	C21H24N2O4	Strictosidine aglycone
	542.248478	7.59785	1.565136212	0.724	0.044	C27H41NO8	Deltaline
	281.0010817	4.5683	1.625882257	1.430	0.038	C10H15OPS2	Fonofos
	195.0631303	4.5683	1.649469396	1.449	0.040	C2H5NO2	Glycine
	277.1446195	6.899216667	2.407843268	0.491	0.011	C6H9N3O	L-Histidinal
	1147.612535	7.462816667	2.756182443	0.394	0.032	C32H46O9	Cucurbitacin A
	606.3386351	7.569266667	1.658559871	0.716	0.038	C37H49NO4	21,22-Diprenylpaxilline
	562.3151158	7.583566667	1.780960894	0.682	0.038	C28H49NO8	Dihydropicromycin
	743.2304057	7.740733333	2.592432786	0.454	0.038	C20H20O7	(1'S)-Averantin
	544.2639155	7.755016667	1.884455548	0.629	0.039	C27H43NO8	Germine
	569.1484759	7.869316667	1.490761831	0.738	0.039	C12H18Cl2NO	Tulobuterol hydrochloride
	279.2322828	7.740733333	2.01333007	0.612	0.024	C18H32O2	Linoleate
	610.2251887	7.626433333	1.235774679	0.817	0.044	C28H39N5O8	Z-Gly-Pro-Leu-Gly-Pro
	632.1819569	7.59785	1.776533892	0.658	0.038	C32H36ClNO8	Clomiphene citrate
	503.2300143	7.583566667	1.95058604	0.608	0.034	C28H38N2O4	Cephaeline
	502.2937389	7.583566667	2.044551487	0.608	0.016	C23H50N2O5P	Sphingosyl-phosphocholine
	636.3141253	7.519966667	2.768383612	0.328	0.044	C37H47NO6	Lolitrem K
	650.2678608	7.462816667	2.04212077	0.600	0.025	C23H45N5O14	6‴-Deamino-6‴-hydroxyneomycin C
	692.2703523	7.462816667	1.987560826	0.635	0.015	C25H47N5O15	2‴-N-Acetyl-6‴-deamino-6‴-hydroxyneomycin C
	557.0683327	7.248533333	1.681498062	0.697	0.038	C14H8O5	Purpurin
	237.0104179	4.5683	1.636732188	1.423	0.038	C9H11ClO3	Chlorphenesin
	173.0815327	4.5683	1.719874482	1.515	0.045	C8H14O4	2-Propylglutaric acid
	366.0818484	3.5775	1.747593046	1.474	0.038	C12H21N5O2S2	Nizatidine

**Fig 5:**
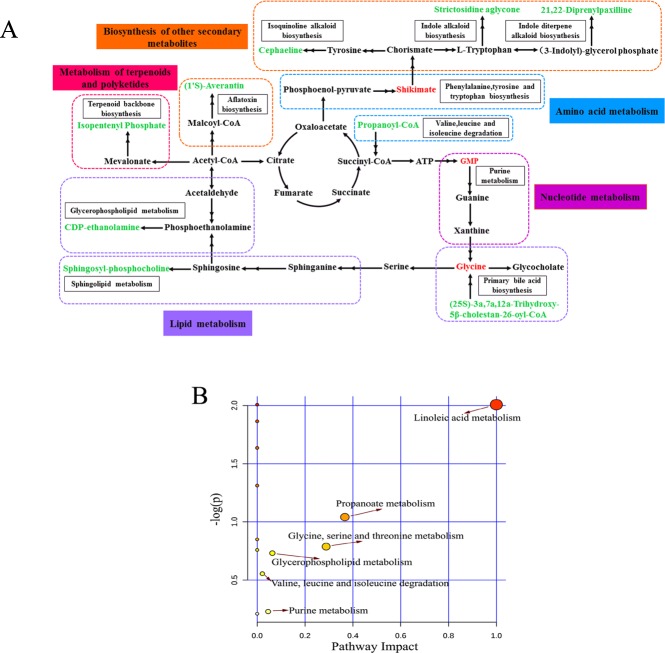
Pathway analysis of the altered metabolites. (A) Schematic overview of the disturbed metabolomic pathways during *Varroa destructor* infestation. Red characters indicate increased metabolites, and green characters indicate decreased metabolites. (B) Summary of pathways analysis with MetaboAnalyst 3.0.

To identify the metabolic pathways disturbed during *V*. *destructor* infestation, the differential metabolites were subjected to KEGG pathway analysis and detailed related pathways were constructed using the reference map deposited in the KEGG database. Of the 25 differentially expressed metabolites detected in ESI- mode, 13 were mapped to the 12 KEGG metabolic pathways. Three metabolites were up-regulated, including GMP, glycine, and 2-propylglutaric acid. Further, of the 39 altered metabolites in ESI+ mode, 10 were mapped to the 10 KEGG metabolic pathways. The numbers of up- and down-regulated metabolites were six and four, respectively. Additionally, as shown in Fig 5A, an integrative view of the metabolic changes was prepared based on our findings. Furthermore, altered pathways based on all metabolites detected in both ion modes were analyzed with MetaboAnalyst 3.0 [15]. The impact value and–log (*P*) with metabolic pathway analysis (MetPA) were carried out to evaluate the importance of the pathways affected during *V*. *destructor* infestation. Among these altered pathways, we identified “linoleic acid metabolism”, “propanoate metabolism”, and “glycine, serine, and threonine metabolism” as three metabolic pathways of interest as all of them exhibited lower *p*-values and greater pathway impact (Fig 5B).

## Discussion

Tissue analysis is perhaps the most powerful approach for studying localized and specific responses to stimuli and pathogenesis and yields explicit biochemical information about the mechanisms of disease. LC-MS-based metabolomic technology has been widely used in the analysis of interactions between pathogens and their hosts [16–18]. The development of *Varroa* populations in honey bee brood cells often induces the death of a colony [19]. Parasitism by the mite causes Asian honey bees to exhibit a series of cleaning behaviors, especially grooming behavior, which can rapidly and effectively kill and remove the mite from the bee hive. However, elucidating the molecular mechanisms underlying this hygienic behavior has been a persistent challenge for clinical and basic researchers. In the present study, LC-MS/MS based on a non-targeted metabolomic method was used to identify the characteristics of the metabolic profile of eastern worker honey bee brains infested with *V*. *destructor*. Our results provide important clues about the molecular metabolic network in the brain during mite infestation, especially the involvement of lipid, carbohydrate, and amino acid metabolism in the nervous system.

Grooming behavior is a crucial strategy for reducing ectoparasites in honey bees. While many studies have explored the mechanisms underlying the hygienic behavior, the molecular bias and functional pathways remain poorly understood. Navajas *et al*. [20] pointed out that the genes involved in neuronal development, neuronal sensitivity, and olfaction determine the susceptibility of honey bees to *Varroa* species. Later, using RNA-Seq, Mondet *et al*. [21] showed that antennae play an important role in the modulation of hygiene behavior, which functions through the alteration of the expression of antennal genes. Another study performed using proteomic methods identified proteins in the antennae of which the expression levels may influence the outcome of mite infestation [22]. Nevertheless, the underlying physiological and neuronal mechanisms remain unclear. Our data from the present study indicate that infestation-specific patterns were acquired in the brain metabolomics profile and that they can be used to distinguish honey bees with mite infestation from the controls. In addition, 64 putative metabolites were identified. Furthermore, by searching these metabolites against the KEGG database, many were found to be involved in metabolic pathways, including lipid metabolism, nucleotide metabolism, and amino acid metabolism amongst others. Integrated network analyses of the metabolites that were differentially expressed during mite infestation yielded highly related signaling networks, strongly suggesting that involvement of these signaling pathways could be essential for the resistance of mite infestation. Further, the metabolic network was mapped using MetaboAnalyst 3.0, and, based on our data, three metabolic pathways—“linoleic acid metabolism”, “propanoate metabolism”, and “glycine, serine, and threonine metabolism” were found to be the most relevant, especially linoleic acid metabolism is significant affected (*p*<0.05). Linoleate is a polyunsaturatedfatty acid, the abundance of which is found decreased in our study. Umezawa *et al*. [23] showed that male senescence-resistant SAMR1 mouse exhibited higher locomotor activity, more active circadian rhythm and higher number of response to stimuli after they were fed food high in linoleate.

The majority of the differentially expressed metabolites detected through both ESI modes decreased in abundance in the infested bees compared to control bees. In the ESI+ mode, iriomoteolide-1a, fenapanil, phytolaccoside B, satratoxin H, and CDP-ethanolamine were most down-regulated with a fold change < 0.5. CDP-ethanolamine is involved in glycerophospholipid metabolism and the CDP-ethanolamine pathway, which is responsible for the *de novo* synthesis of phosphatidylethanolamine [24]. In humans, phosphatidylethanolamines are found particularly in nervous tissue such as the white matter of brain, nerves, neural tissue, and in spinal cord, where they make up 45% of all phospholipids [25]. Meanwhile in the ESI- mode, another four metabolites exhibited two times the down-regulated expression levels, including lolitrem K, cucurbitacin A, (1'S)-averantin, and L-histidinal. L-histidinal, a biosynthetic precursor of histidine, is involved in histidine metabolism and the biosynthesis of secondary metabolites [26]. In addition to the most altered metabolites, there are a few others worthy of mentioning. For example, GMP is a precursor for cyclic GMP (cGMP) through the action of phosphodiesterase (PDE), and our results showed that the level of GMP increased with mite infestation, which may function through the involvement of purine metabolism and hormonal signaling [27,28]. A previous study showed that GMP along with other guanine-based purines exhibited important neruromodulatory function [29]. In rat, acute administration of GMP was able to decrease the levels of anxietyin classical behavioral tasks [30]. Sphingolipids are highly bioactive compounds involved in diverse cell processes including cell-cell interactions and cell proliferation, differentiation, and apoptosis [31]. In this study, sphingosyl phosphochine was up-regulated in the sphingolipid metabolism pathway, suggesting sphingolipids may be an intracellular second messenger to function. Moreover, the alternation of sphingosyl phosphochine associated with *Varroa*-defense behavior mechanisms has not been reported; thus, further studies are needed to elucidate these.

Rhythmic behaviors—such as walking, hygienic behavior, grooming, and flying—are controlled by central pattern generators [32]. Grooming behavior significantly increased in bees infested by mites, indicating that some substances may act on neurons involved in pattern generation. Mosapride citrate hydrate has been recognized as a 5-HT_4_ agonist, which exhibited decreased expression levels in this study. 5-HT_4_ is a glycosylated transmembrane protein that functions in both the peripheral and central nervous systems to modulate the release of various neurotransmitters [33]. Isopentenyl phosphate (IPP), a down-regulated metabolite in the mevalonate pathway, functions as a dual inhibitor for transient receptor potential (TRP) A1 and TRPV3. Bang *et al*. [34] showed that peripheral IPP could administer attenuated TRPA1 and TRPV3 agonist-specific acute pain behaviors. Securinine, exhibiting a decreased level in infested bees compared to control bees in our study, serves as a selective antagonist of GABA recognition sites on mammalian central neurons, and it can inhibit GABA binding to rat brain membranes [35].

In conclusion, changes in the metabolic profiles of eastern honey bee brains exposed to *V*. *destructor* infestation were observed by a high-throughput non-targeted metabolomics method. Sixty-four putative significantly changed metabolites were identified, and the major metabolite network was predicted by pathway analysis. The putative metabolites were involved in a variety of pathways related to amino acid metabolism, nucleotide metabolism, and lipid metabolism. Worthy to mention, previous studies showed that the tactile and olfactory aspects of the mite were closely related to the bee defense behavior. However, the current study doesn’t distinguish between tactile and olfactory cues that may be important for eliciting grooming behavior in bees. This will be our next study focus. The differential metabolites and related pathways identified not only further our understanding of the metabolic responses of honey bees to *Varroa* parasitism but also provide important molecular clues involved in the signaling cascades regulating hygienic behaviors. Also worth mentioning is that *A*. *mellifera* is susceptible to the *V*. *destructor* mite, frequently resulting in colony disintegration. Our present work lays a solid foundation for broadened the understanding of western honey bee metabolic profiles after mite infestation.

## Materials and method

### Honey bee colonies

The *A*. *cerana* populations were reared at the Apicultural Research Institute, CAAS, Beijing, China. Three honey bee colonies with mated queens of the same identical age and similar colony strength were selected as the experimental colonies.

### Mite collection

Adult *V*. *destructor* mites were collected from a highly infested *A*. *mellifera* colony. Firstly, 50 adult *A*. *mellifera* were collected from hive frames and then transferred into a wire cage (8.7×6.0 cm), which was covered with a 4.0 mm wire mesh screen. Next, the wire cage was placed in a sealed container where the bees and mites were anesthetized with CO_2_ for 2 min, allowing the mites to fall off from the worker bees. The mites were collected from the bottom of the container, placed in a petri dish, and kept at room temperature in the laboratory. This mite collection method was referred to in Arechavaleta-Velasco et al.’s study [36].

### Observation hive

The observation hive used in this study was designed according to the method described by Arechavaleta-Velasco *et al*. [36] with slight modifications. Briefly, the observation hive was built out of wood (45×16×3 cm), and one end was covered with a piece of wire mesh (16×10 cm) for ventilation. The rest of the hive was covered with a transparent clear plastic sheet 1 mm thick. In the center of the plastic sheet, a circular hole 9 cm in diameter was made, which allowed the Plexiglas^®^ cage (observe cage) to be inserted into the hive.

### Behavioral assays

Approximately 1000 worker bees (*A*. *cerana*) in a comb along with one queen from a colony were firstly placed in the observation hive, which was then sealed and taken to the laboratory [36]. The schematic of the observation hive is shown in S3 Fig. Grooming behavior is defined as swiping motions in the direction of the mite with the front two pairs of legs. Grooming behavior at the individual level was evaluated as previously described [37]. Ten to 11-day-old worker honey bees were used in this study, and bees at this age show strongest grooming response. A total of 300 bees were divided into infestation group and control group, each group consists of 10 replicates. Each replicate having 15 bees, and these bees were equally selected from the three colonies. Bees in infestation group were selected and anesthetized with CO_2_ to allow for manipulation of the bees. Then each bee was introduced into a small (10 × 10 × 7 cm) modified Plexiglas^®^ cage. The Plexiglas cage has small pores (diameter ≤0.3 cm) on its wall, through which the tested bees can not walk out. When a bee appeared to have fully recovered, a *V*. *destructor* mite was placed on her thorax using a fine brush. The observation assays were performed in a dark environment with a red light on. Grooming behaviors were recorded with a video camera in 5 minutes. As for the control group, use the same method except for infested *Varroa destructor*. A Student’s *t*-test was conducted to test for statistical differences in grooming behavior between control and infested mites; *p*-values < 0.05 were deemed statistically significant.

### Sample collection

Another 300 honey bees were collected from the same test colonies and divided into two groups: *V*. *destructor*-infested and control groups, 10 replicates in each group. Each replicate having 15 bees, and these bees were equally selected from the three colonies. Using the same method, observation was performed on bees grooming behaviors for 5 min before being flash-freezing in liquid nitrogen.

### Brain dissection and metabolite extraction

Using a dissecting microscope, bee brains were dissected on dry ice to remove all traces of the optic lobe and then stored at −80°C until further processing. A total of 25 mg of brain samples was homogenized in 80 μL of a precooled methanol/water (1:1) solvent and ground for 5 min. The mixture was centrifuged at 20 000 ×g for 10 min at 4°C. Supernatant (200 μL) was transferred to a new 1.5 mL polypropylene tube and processed through vacuum freeze drying before liquid chromatography separation.

### UPLC-MS/MS analysis

Liquid chromatography was performed on a 2777C UPLC system (Waters, UK). The separation of all samples was performed on an ACQUITY UPLC^®^ BEH C18 column (Waters, UK) (100 mm × 2.1 mm, 1.7 μm). A gradient elution program was run for chromatographic separation with mobile phase A (water), and mobile phase B (acetonitrile) as follows: 0~2 min, 100% A-100% A; 2~12 min, 100% A-0% A; 12~14 min, 0% A-0% A; and 14~15 min, 0% A-100% A. The injection volume was 10 μL, and the flow rate was set to 0.4 mL/min. A SYNAPT G2 XS QTOF (Waters, U.K.) equipped with an electrospray ionization (ESI) source was used for mass spectrometric detection. Sample analyses were performed in both positive and negative ion modes. The operating parameters were as follows: capillary, 0.3 kV (ESI+) or 2 kV (ESI-); sampling cone, 40 V; source temperature, 120°C (ESI+) or 100°C (ESI-); desolvation temperature, 500°C (ESI+) or 350°C (ESI-); desolvation gas, 800 (L/h); cone gas, 50 (L/h); source offset, 80; TOF acquisition mode, sensitivity (ESI+) or sensitivity (ESI-); acquisition method, continuum MS^E^; TOF mass range, 50–1200 Da; scan time: 0.1 s; and collision energy function 2, trap CE ramp 20–40 eV.

### Data processing

For quantitative metabolomics, raw data files were uploaded into Progenesis QI software (version 2.1), within which data alignment, normalization, and peak picking were performed. Next, the data matrix was mean-centered, pareto-scaled, and analyzed by both principal component analysis (PCA) and partial least squares discriminant analysis (PLS-DA). The quality of the models was evaluated with the relevant R^2^ and Q^2^ as described elsewhere [38]. Meanwhile, permutation tests (1000 cycles) were conducted to assess the robustness of the PLS-DA model. Differential metabolites were selected when the statistically significant threshold of variable influence on projection (VIP) values obtained from the PLS-DA model was larger than 1.0. Meanwhile, differences were tested with a Student’s *t*-test, and a corrected *p*–value (*q*-value) < 0.05 was deemed statistically significant. Log_2_ fold change (FC) was used to show how these selected differential metabolites varied between groups. A heat map was generated using the MultiExperiment Viewer (MeV) v.4.9 software (http://www.tm4.org/mev.html) based on the abundance of differentially expressed metabolite data (log_2_-scaled) [39]. To check and confirm the putative differentially expressed metabolites, the HMDB and KEGG databases were used [40, 41]. Candidate metabolites were confirmed by MS/MS scans for the characteristic ions and fragmentation patterns of the compound. A pathway analysis of the differentially expressed metabolites was performed based on the HMDB and KEGG databases. MetaboAnalyst (http://www.metaboanalyst.ca) is a comprehensive web application for metabolomic data analysis and interpretation [15]. For the pathway enrichment analysis, a hypergeometric test was used for the over representation analysis, and relative-betweenness centrality was used for the pathway topology analysis. In the MetaboAnalyst analysis, a *p*-value of 0.05 was set as the threshold for significance.

## Supporting information

S1 Fig **Overlay of all total ion current (TIC) chromatograms for serum samples obtained in the (A) positive ion mode (ESI+) and (B) negative ion mode (ESI-).** The y-axis represents the intensity.
(TIF)

S2 Fig PCA of bee brain samples. (A) Score plot for the positive ion mode; (B) Score plot for the negative ion mode.
(TIF)

S3 Fig The schematic of the observation hive. (TIF)
