# Supplementary figures and images for: Brain metabolomic profiling of eastern honey bee (Apis cerana) infested with the mite Varroa destructor
Source: PLoS One. 2017 Apr 12;12(4):e0175573. doi: 10.1371/journal.pone.0175573 (PMC5389839; doi:10.1371/journal.pone.0175573)

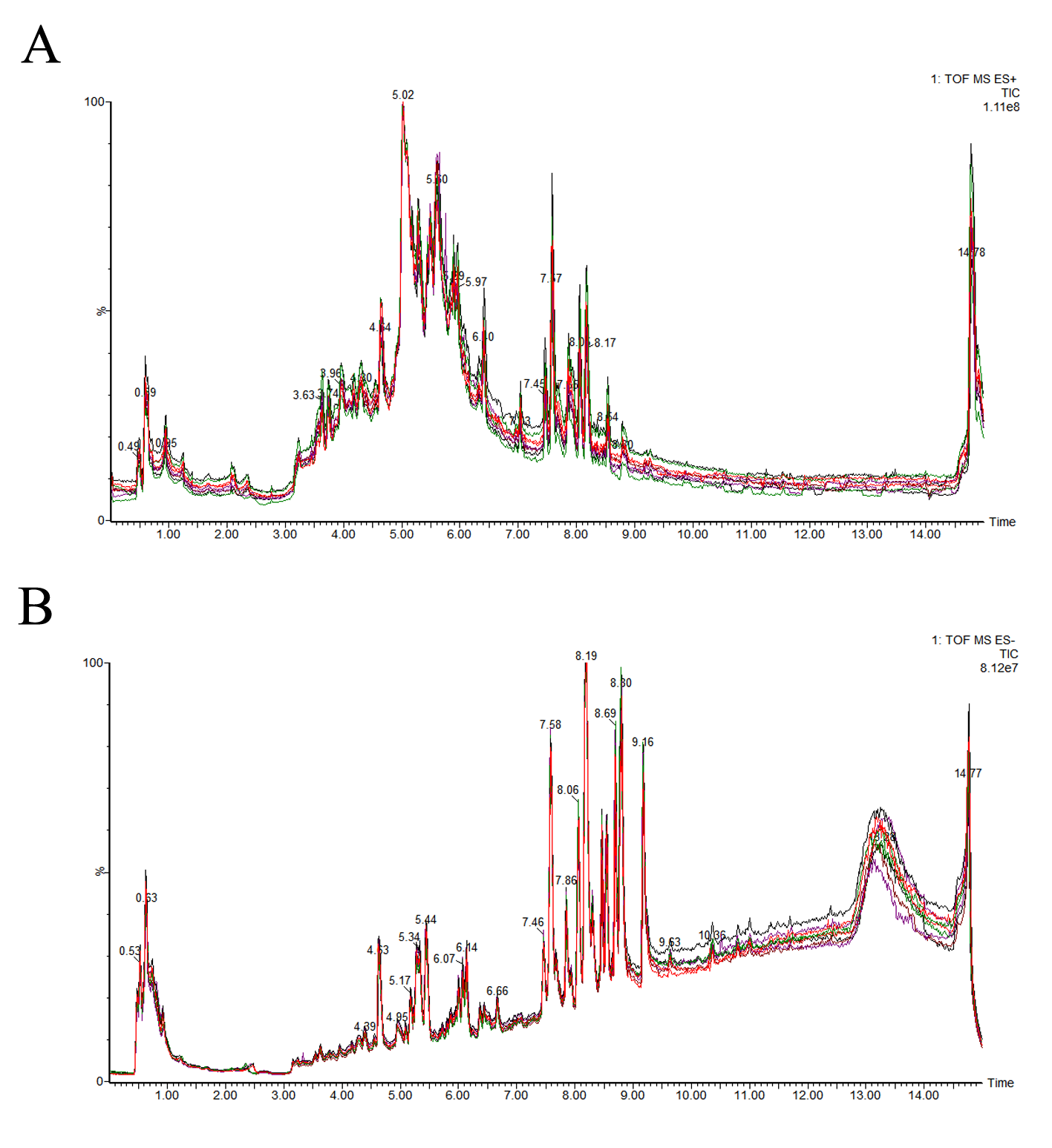

Supplement: S1 Fig — Overlay of all total ion current (TIC) chromatograms for serum samples obtained in the (A) positive ion mode (ESI+) and (B) negative ion mode (ESI-). The y-axis represents the intensity. (TIF) [file pone.0175573.s001.tif]

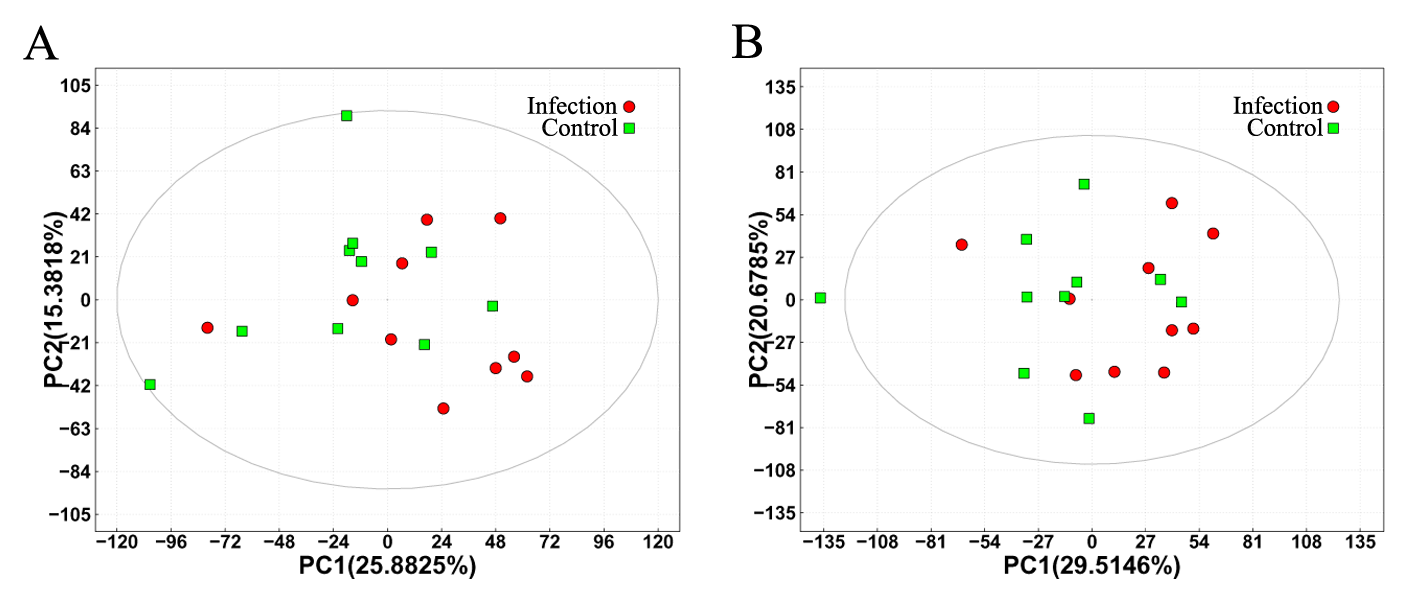

Supplement: S2 Fig — (A) Score plot for the positive ion mode; (B) Score plot for the negative ion mode. (TIF) [file pone.0175573.s002.tif]

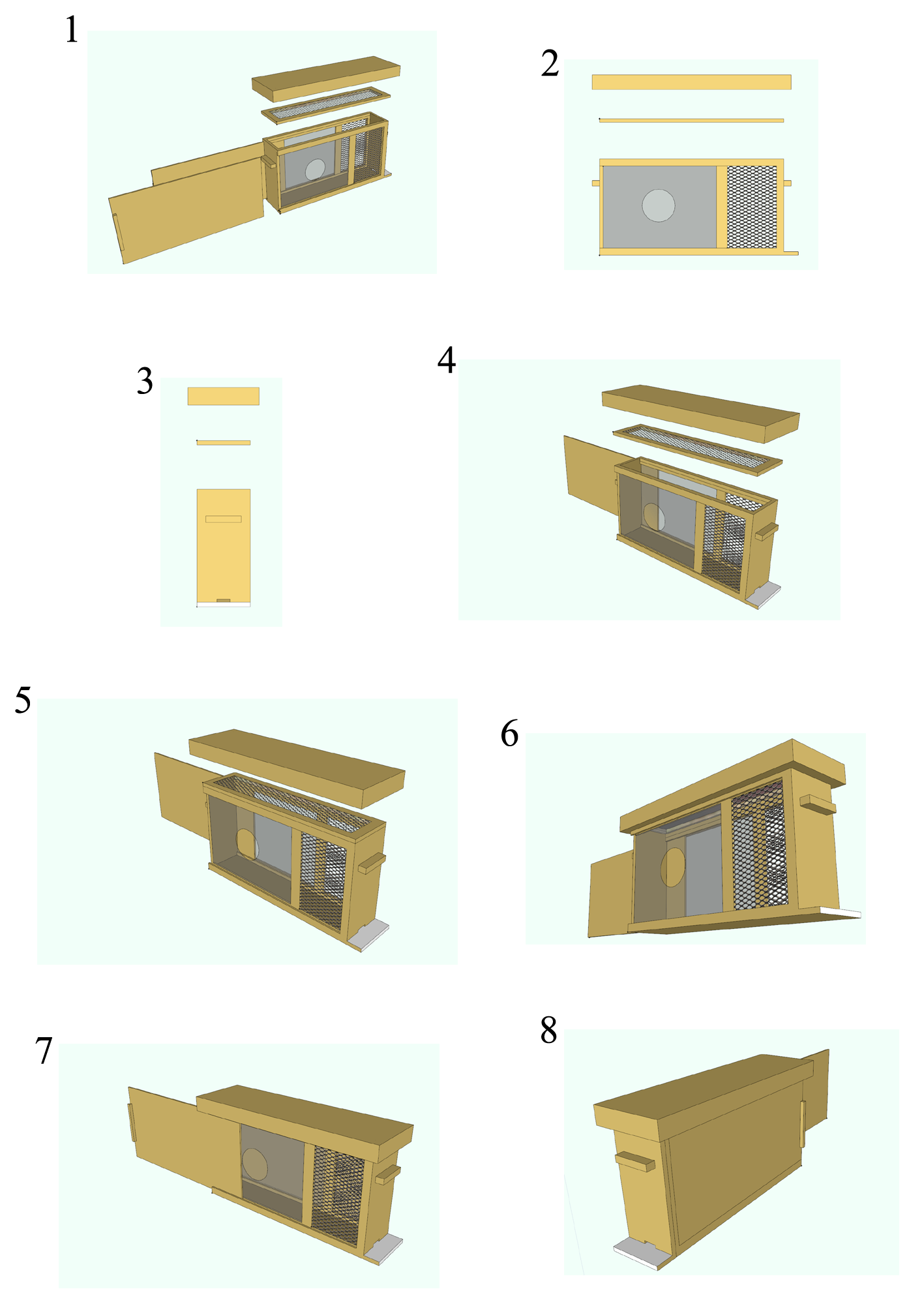

Supplement: S3 Fig — (TIF) [file pone.0175573.s003.tif]
